# A cell-nonautonomous mechanism of yeast chronological aging regulated by caloric restriction and one-carbon metabolism

**DOI:** 10.1074/jbc.RA120.015402

**Published:** 2020-12-02

**Authors:** Elisa Enriquez-Hesles, Daniel L. Smith, Nazif Maqani, Margaret B. Wierman, Matthew D. Sutcliffe, Ryan D. Fine, Agata Kalita, Sean M. Santos, Michael J. Muehlbauer, James R. Bain, Kevin A. Janes, John L. Hartman, Matthew D. Hirschey, Jeffrey S. Smith

**Affiliations:** 1 Department of Biochemistry and Molecular Genetics, University of Virginia School of Medicine, Charlottesville, Virginia, USA; 2 Department of Nutrition Science, Nathan Shock Center of Excellence in the Basic Biology of Aging, University of Alabama at Birmingham, Birmingham, Alabama, USA; 3 Department of Biomedical Engineering, University of Virginia School of Medicine, Charlottesville, Virginia, USA; 4 Department of Genetics, Nutrition and Obesity Research Center, Nathan Shock Center of Excellence in the Basic Biology of Aging, University of Alabama at Birmingham, Birmingham, Alabama, USA; 5 Department of Medicine, Duke Molecular Physiology Institute, Duke University, Durham, North Carolina, USA

**Keywords:** *Saccharomyces cerevisiae*, caloric restriction, aging, chronological life span, cell-nonautonomous, serine, one-carbon metabolism, amino acids, AMPK, AMP-activated protein kinase, BCAA, branched chain amino acids, CFUs, colony forming units, CR, caloric restriction, CLS, chronological life span, CRCM, CR conditioned media, GAAC, general amino acid control, NR, nonrestricted, QTL, quantitative trait locus, RT, retention time, SC, synthetic complete, TCA, tricarboxylic acid cycle, TOR, target of rapamycin

## Abstract

Caloric restriction (CR) improves health span and life span of organisms ranging from yeast to mammals. Understanding the mechanisms involved will uncover future interventions for aging-associated diseases. In budding yeast, *Saccharomyces cerevisiae*, CR is commonly defined by reduced glucose in the growth medium, which extends both replicative and chronological life span (CLS). We found that conditioned media collected from stationary-phase CR cultures extended CLS when supplemented into nonrestricted (NR) cultures, suggesting a potential cell-nonautonomous mechanism of CR-induced life span regulation. Chromatography and untargeted metabolomics of the conditioned media, as well as transcriptional responses associated with the longevity effect, pointed to specific amino acids enriched in the CR conditioned media (CRCM) as functional molecules, with L-serine being a particularly strong candidate. Indeed, supplementing L-serine into NR cultures extended CLS through a mechanism dependent on the one-carbon metabolism pathway, thus implicating this conserved and central metabolic hub in life span regulation.

Caloric restriction (CR) extends life span in a wide variety of model organisms ranging from the budding yeast, *Saccharomyces cerevisiae*, to nonhuman primates, implying that conserved cellular processes and pathways must mediate the beneficial effects or somehow be impacted by the dietary regimen (1). Indeed, conserved processes including autophagy, target of rapamycin (TOR) signaling, and AMP-activated protein kinase (AMPK) signaling have each been implicated in regulating aging in most models of CR (2). In the yeast system, CR is typically characterized by reducing the initial glucose concentration in growth medium from 2% (nonrestricted; NR) to 0.5% or lower, or reducing overall amino acids (3, 4). Glucose restriction robustly extends both yeast replicative life span (RLS) and chronological life span (CLS) (3, 4, 5, 6), the latter of which is defined by the number of days that nondividing cells maintain proliferative capacity in liquid culture after entering stationary phase, quantified upon transfer to fresh nutrient media (7, 8). As glucose becomes limiting toward the end of exponential growth, cells switch from fermentative (metabolism of glucose to ethanol) to mitochondrial-driven oxidative metabolism of the ethanol and organic acids produced during fermentation. This “diauxic shift” is accompanied by dramatic changes in transcription, translation, and metabolic profiles that facilitate slower cell growth using nonfermentable carbon sources (9, 10), ultimately leading to cell cycle exit and quiescence. CLS largely hinges on an adaptive response to nutrient depletion, consisting of cell cycle exit (G0), called quiescence (11, 12). Yeast CLS is therefore considered a model for the aging of quiescent stem cells or postmitotic cells such as neurons and muscle fiber cells (13, 14, 15).

Since the initial glucose concentration in yeast cultures has profound impact on long-term cell survival, fully understanding intracellular and extracellular responses underlying the complex adaptive transition into quiescence, and how CR influences them, is of central interest. CR enhances several processes that occur during the diauxic shift, including Snf1 (AMPK) signaling (16), mitochondrial respiration and ATP production (6, 17, 18, 19), accumulation of the storage carbohydrate trehalose (20), and improved G1 cell cycle arrest (21). In addition to CR, there are several other conserved genetic and environmental manipulations that extend CLS, including inhibition of TOR signaling (22) and methionine restriction (23, 24).

As a unicellular organism, the impact of environmental and genetic modifications on budding yeast survival is usually expected to occur through cell-autonomous mechanisms such as direct changes in gene expression, metabolism, and stress response. However, regulation of survival by genetic and environmental manipulations is also linked with cell-nonautonomous mechanisms driven by cell-derived extracellular factors (25, 26). For example, acetic acid released by cells during CLS assays accumulates and acidifies the media. The resulting acetic acid stress response activates nutrient sensing pathways that lead to elevated superoxide (27), apoptosis, and reduced life span (25). Importantly, acetic acid accumulation is suppressed by CR or TOR inhibition (25, 28), thus contributing to the beneficial effects on CLS. Buffering the media pH or transferring cells to water after stationary phase also effectively protects yeast cells from this cell-nonautonomous aging mechanism (25, 29).

Longevity-associated cell-nonautonomous mechanisms are classically described from rodent models, where circulating extracellular factors have been identified from heterochronic parabiosis experiments (30). For example, mesencephalic astrocyte-derived neurotrophic factor (MANF) from a younger mouse protects against liver damage in the older mouse (31). MANF overexpression also extends life span in *Drosophila* (31). Interestingly, some factors that act in a cell autonomous manner, such as the insulin-like signaling transcription factor FOXO, can impact organismal longevity *via* cell-nonautonomous mechanisms (32, 33), raising the possibility that such processes are more widespread than previously thought.

Despite being single cell organisms, budding yeast utilizes proteins and metabolites for cell–cell communication associated with mating, differentiation, and sporulation. Recognition of opposite haploid mating types (a-cells or α-cells) occurs *via* the extracellular pheromone peptides a-factor and α-factor (34), whereas pseudohyphal growth in dense cultures or colonies is mediated by quorum sensing *via* the amino acid–derived aromatic alcohols, tryptophol, and phenylethanol (35). Chronological aging of *S. cerevisiae*, which occurs in densely crowded cultures and is highly sensitive to gene–nutrient interactions (36), would also seem subject to cell-nonautonomous mechanisms. Indeed, unidentified high-molecular-weight (>5000 Da) extracellular factors from old stationary phase cultures have been implicated in stimulating survival of other old cells (37). Similarly, our lab observed that conditioned media isolated from glucose-restricted stationary phase cultures, referred to as CR conditioned media (CRCM) throughout this current study, extended CLS when supplemented into NR cultures (38), suggesting the presence of one or more extracellular proteins, peptides, or metabolites that contribute to life span regulation. We were interested in elucidating these factors in order to provide new insights about CR mechanisms. To this end, we utilized a combination of chromatography, metabolomics, and targeted mass spectrometry to identify functional candidate factors that were more abundant in CRCM than conditioned media from NR cultures (NRCM). Longevity activity was traced to multiple amino acids that extend CLS when supplemented into NR cultures. We focused further analysis on L-serine and, in the process, implicated the one-carbon metabolism pathway in CLS regulation.

## Results

### Conditioned media from CR stationary-phase cultures contains longevity factors

**Figure 1:**
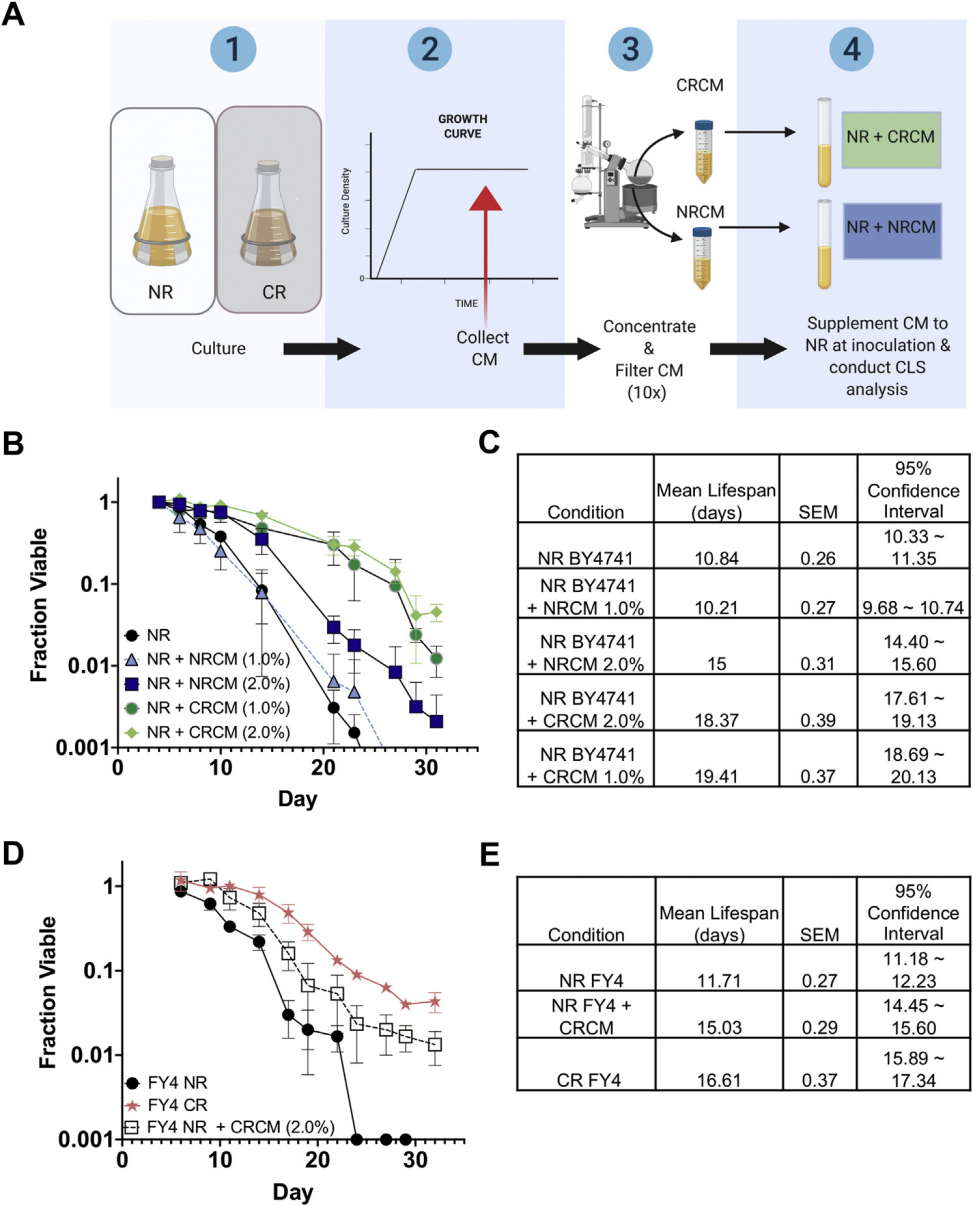
**Longevity factors are present in conditioned media from calorie-restricted yeast cultures.***A*, schematic of conditioned media CLS experiments. BY4741 was grown to stationary phase in SC media containing 2% (NR) or 0.5% (CR) glucose. Cells were pelleted by centrifugation and the conditioned media (CRCM or NRCM), then concentrated tenfold with a Büchi Rotavap apparatus, and supplemented into small 15 ml NR cultures of BY4741 or FY4. *B*, quantitative chronological life span (CLS) assay. Concentrated CRCM and NRCM were supplemented at 1% or 2% (vol/vol) into NR cultures at time of inoculation. To measure the fraction of viable cells over time, microcolony forming units were counted after 18 h of regrowth after spotting onto YPD plates. Error bars indicate standard deviations (n = 3). *C*, mean life span in days was calculated using Online Application for Survival Analysis Software (OASIS 2), which reports standard error of the mean (SEM) and 95% confidence intervals. *D*, quantitative CLS assay showing effect of CRCM at 1% (vol/vol) on prototrophic strain FY4. *E*, statistical analysis of results from panel D. All CLS assays had three biological replicates.

To further investigate the extracellular mechanisms underlying CR-induced CLS extension, we collected and concentrated CRCM and NRCM from day 5 stationary-phase cultures of BY4741, a commonly used lab strain (Fig. 1*A*). Each concentrate was then titrated into NR SC media at 1:10, 1:20, or 1:50 ratios. We reasoned that using concentrated media would increase the chances of detecting longevity activity if the factors were low-abundance molecules. CLS of BY4741 was initially analyzed using a 384-well quantitative high-throughput cell array phenotyping (Q-HTCP) system that allowed 96 replicates for each condition (Fig. S1*A*). With Q-HTCP, improved CLS is indicated by a lower L parameter on the y-axis, representing the time it takes a small aliquot spotted onto a fresh YPD plate to reach ½ maximum growth density (36, 39). If more viable cells are spotted, then it takes less time to reach ½ max growth. Compared with the control (dH_2_O), CRCM supplementation clearly lowered the L parameter in a dose-dependent manner (Fig. S1*A*), corresponding to extended life span in survival curves (Fig. S1*B*). In contrast, NRCM only had minor beneficial effects at the higher (1:10 and 1:20) supplementation levels, suggesting that significantly higher concentrations of putative extracellular longevity factors were present in the CRCM. We also tested whether further dilution of the CRCM concentrate would extend CLS when grown in larger 10 ml cultures and quantified using our standard microcolony viability assay (see (16) and Experimental Procedures). As shown in Figure. 1, *B* and *C*, the CRCM concentrate significantly extended CLS of BY4741 when diluted 1:50 (2% vol/vol) or 1:100 (1% vol/vol) in NR SC media, while NRCM concentrate had a minor effect only at 2% (vol/vol). Since BY4741 is auxotrophic for histidine, leucine, methionine, and uracil, we next confirmed that concentrated CRCM isolated from this strain could also extend the CLS of a prototrophic strain, FY4, when supplemented into NR SC (Fig. 1, *D* and *E*). We concluded that the putative longevity factors were significantly more abundant in CRCM than NRCM.

### Fractionation of CRCM isolates CLS factor activities separate from acetic acid

**Figure 2:**
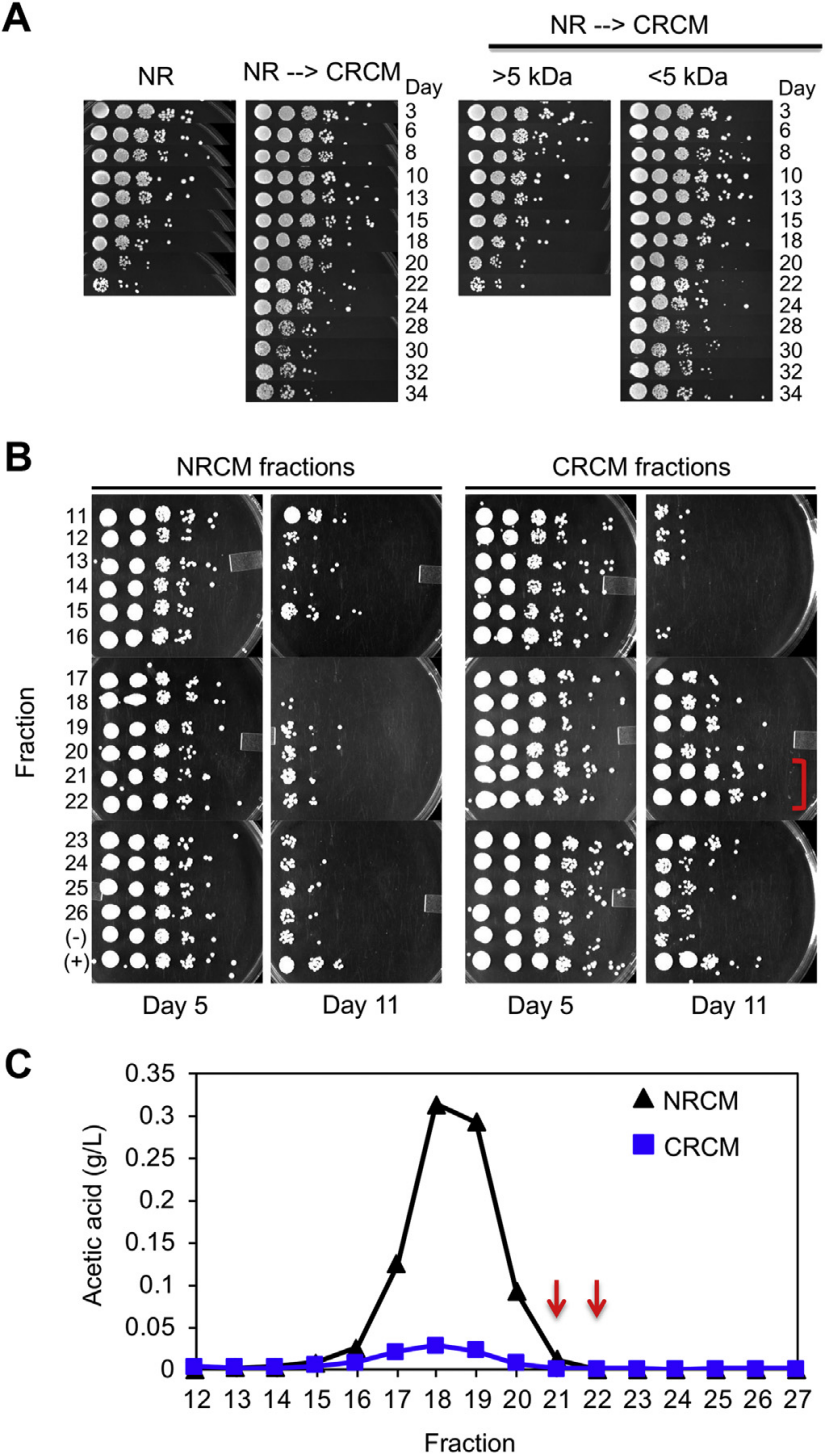
**Chromatographic sizing and separation of longevity factor activity in CRCM.***A*, Left panel: Qualitative CLS assay showing improved viability when supplementing CRCM into nonrestricted BY4741 cultures. Right panel: CRCM was first separated into high MW (>5 kDa) or low MW (<5 kDa) fractions using an Amicon Ultra-4 centrifugal filter unit, then supplemented into nonrestricted BY4741 cultures. Longevity activity was retained in the low MW fraction. *B*, size-exclusion chromatography of NRCM and CRCM was performed using a Sephadex G-10 column (700 Da MW cutoff). Fractions were added to nonrestricted BY4741 cultures at the time of inoculation, and viability tracked over time with qualitative spot assays. The effects of fractions 11–26 are shown for days 5 and 11. The longevity peak fractions for CRCM are bracketed in red. *C*, acetic acid concentration (g/L) was measured for each fraction. *Red arrows* indicate the fractions (21 and 22) with longevity activity in CRCM.

The preliminary experiments in Figures 1 and S1 provided evidence of titratable longevity factors enriched in the media of CR cultures, thus raising the question of their chemical nature and identities. An earlier study concluded that chronologically aged yeast cells release large (>5 kD) heat-stable compounds into the media that improve viability of other cells in the population (37). To determine if CRCM contained such factors, we treated it with Proteinase K, DNase I, RNase A, phenol/chloroform, autoclaving, or freezing, but none of these had any effect on CLS extension (data not shown). Instead, CRCM activity was found to be smaller than 5000 Da, as the fraction passing through an Amicon Ultra-4 centrifugal filter unit (5000 MW cutoff) had CLS extending activity equivalent to the starting material (Fig. 2*A*). This result demonstrated that the CR-associated longevity factors described here were different from the previously described higher-molecular-weight factors (37).

To more effectively size the active CLS-modifying molecules in conditioned media using size-exclusion chromatography, we rigorously concentrated 150 ml of NRCM or CRCM down to a final volume of 2.5 ml (60-fold). Precipitates were then removed by centrifugation, and the remaining soluble material fractionated through a Sephadex G-10 column, which has a size-exclusion limit of ∼700 Da. Fractions were added to NR SC cultures of BY4741 at a 1:5 ratio and CLS extension detected using a qualitative spot test assay (Fig. 2*B*). At day 11, there was a clear peak of improved viability at fractions 21 and 22 for the CRCM, suggesting that the active compounds were smaller than 700 Da (Fig. 2*B*). We considered the possibility that high levels of acetic acid in the NRCM could potentially mask longevity activity in the fractions. However, acetic acid peaked at fractions 18–19 in these columns, distinct from the CRCM longevity peak at fractions 21–22 (Fig. 2*C*, *red arrows*). Instead, the reduced day 11 viability with NRCM fractions 16–18 was potentially due to elevated acetic acid (Fig. 2, *B*–*C*). Based on this size-exclusion chromatography and the resistance to various treatments such as heat, phenol extraction, nuclease digestion, etc., we concluded that the longevity factors in CRCM were small water-soluble compounds separable from acetic acid.

### CRCM-enriched metabolites and induced genes indicate amino acids modulate CLS

**Figure 3:**
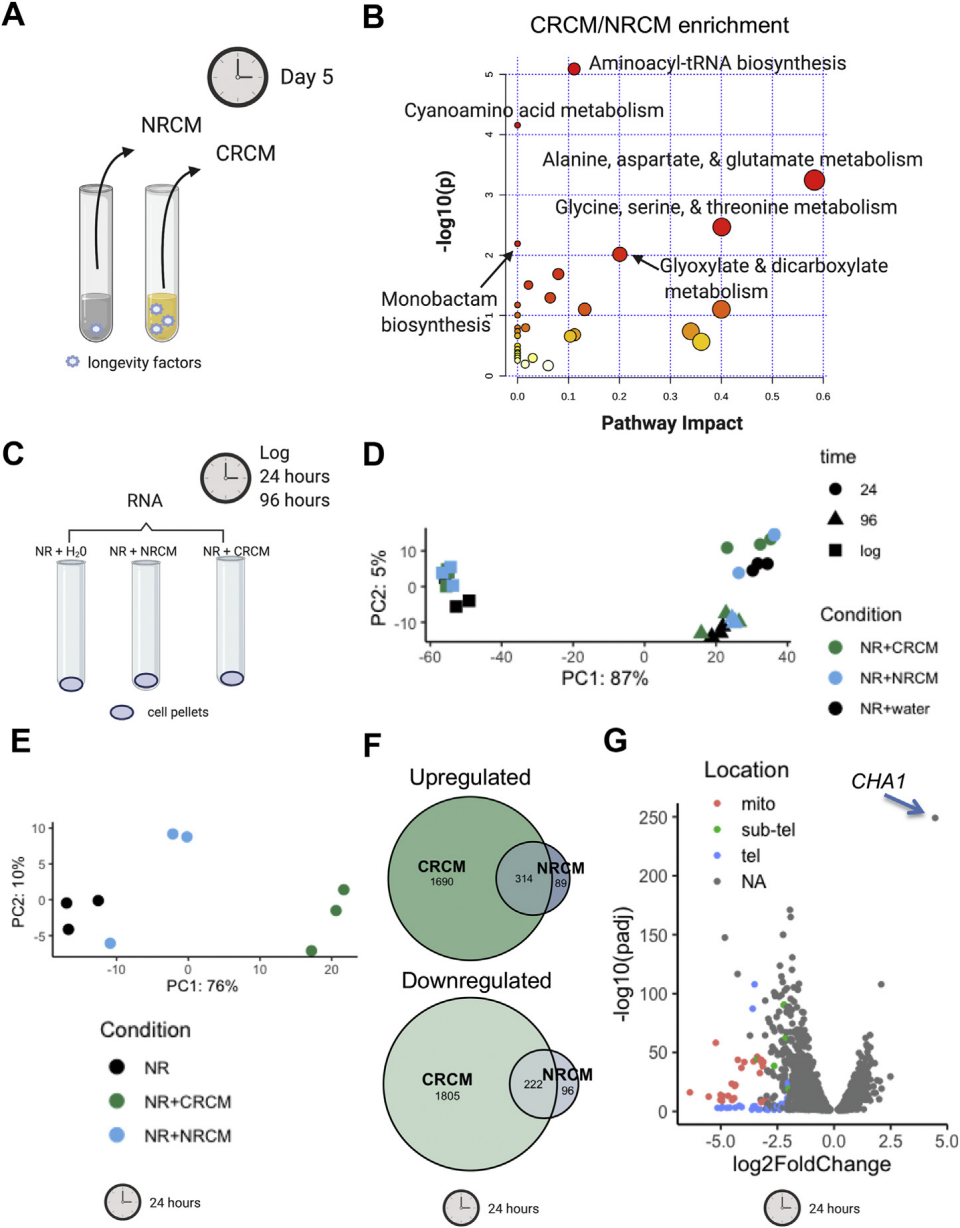
**Metabolomic and RNA-seq analysis point toward amino acid metabolism.***A*, schematic of conditioned medium collection used for untargeted metabolomics analysis after 5 days of SC cultures grown with 2.0% (NR) or 0.5% glucose (CR). *B*, metaboAnalyst software was used for Enrichment and Pathway Analysis of metabolites with a CR/NR ratio greater then 1.0. The names of KEGG pathways with *p* < 0.05 are highlighted. Pathway impact is a measure that considers the centrality of a metabolite in the pathway. Circle size is proportional to pathway impact value, and *darker red color* indicates more significant changes. *C*, schematic of cell conditions (NR, NR + 2.0 % NRCM, and NR + 2.0% CRCM) collected for RNA-seq analysis at 6 h (log phase), 24 h, and 96 h. Three biological replicates were performed for each condition. *D*, principal component analysis of RNA-seq samples at log, 24, and 96 h conditions of NR, NR+ NRCM, and NR+ CRCM. *E*, principal component analysis of RNA-seq samples collected at 24 h. *F*, Venn diagram of differentially expressed genes (up or down; FDR<0.05) for NRCM- or CRCM-supplemented samples, as compared with the NR + H_2_O control at 24 h. *G*, volcano plot displaying differential expressed genes between the NR + CRCM and NR + H_2_O samples at 24 h. The y-axis indicates the p-adjusted value, and x-axis the log2 fold change. *Red*, *green*, and *blue* denote genes located in the mitochondrial genome, subtelomeric, or telomeric regions, respectively. The most upregulated gene *CHA1* is highlighted by an *arrow*.

Knowing that the extracellular longevity factors were small molecules, we next utilized a comparative metabolomics approach to generate metabolite profiles for the CR and NR conditioned media, reasoning that differential abundance of extracellular metabolites could be a source of CRCM longevity factors (Fig. 3*A*). Enrichment and pathway analysis of metabolites more abundant in the CRCM compared with NRCM (Table S1) was performed using MetaboAnalyst (40), in which pathway impact (relative circle size) is a measure that considers the centrality of a metabolite in the pathway (Fig. 3*B*). The most significantly enriched categories (shaded red) were related to amino acids, including L-alanine, L-aspartate, and L-glutamate metabolism, as well as L-glycine, L-serine, and L-threonine biosynthesis, both of which had the two highest pathway impact scores (Fig. 3*B*).

To gain additional insights about candidate factors that were potentially functional, we also performed transcriptomics analysis on BY4741 cells grown in NR SC media supplemented with CRCM, NRCM, or water as a control (Fig. 3*C*), with a goal of identifying physiological responses linked to specific metabolites. Following inoculation into these conditions, we harvested cells at 6 h (log phase), 24 h (late diauxic shift), and 96 h (stationary phase), then performed RNA-seq on isolated mRNAs from three biological replicates. Principal component analysis (PCA) indicated the major variance within the data was the time points (Fig. 3*D*), consistent with the massive transcriptional changes that occur during the transition into stationary phase (9, 10). In early log-phase cells, there were no significantly upregulated or downregulated genes in the CRCM- or NRCM-supplemented samples as compared with the H_2_O-supplemented control (FDR <0.05), consistent with earlier microarray analysis showing that CR (0.5% glucose) had little effect on gene expression during early log phase (38). At the 24 h time point, however, CRCM-supplemented samples diverged from the NRCM- and H_2_O-supplemented controls in a PCA plot (Fig. 3*E*) and showed many more differentially regulated genes than the NRCM-treated samples (Fig.3*F*). At the 96 h time point, gene expression for the NRCM samples also clearly differentiated from the H_2_O-supplemented control (Fig. S2*A*), though there were still a large number of genes exclusively altered in the CRCM samples (Fig. S2*B*). The top GO term for CRCM-upregulated genes at 96 h was α-amino acid catabolic process (Table S2), consistent with the MetaboAnalyst results indicating amino acid metabolism. Interestingly, there were a number of telomeric and subtelomeric ORFs that were more tightly repressed in the CRCM-treated cells compared with the NR control at 24 and 96 h (Fig. 3*G*, S2*C*, and Tables S4, S5), suggesting that the general transcriptional repression associated with chromatin condensation in quiescent cells may be enhanced by supplementing with CRCM (41, 42). At 24 h, the top GO terms for CRCM-upregulated genes were related to mitochondrial function and respiration, consistent with a more robust metabolic transition during the diauxic shift (Table S3). Furthermore, the *YCL064C* (*CHA1*) gene, which is adjacent to the heterochromatic *HML* locus, clearly stood out as the most significantly upregulated (Fig. 3*G* and Table S4). *CHA1* encodes a predominantly mitochondrial L-serine (L-threonine) deaminase that catabolizes these amino acids as nitrogen sources and, in the case of L-serine, for direct production of pyruvate (43, 44). It is strongly upregulated by exogenous L-serine or L-threonine added to the growth medium (43, 45), suggesting that L-serine and possibly L-threonine in the CRCM could be producing an especially strong physiological response related to CLS extension. Together, the extracellular metabolite analysis and effects on gene expression during the diauxic shift and stationary phase pointed toward amino acids, especially L-serine, as candidate extracellular CLS factors mediating the CR effect on CLS.

### Amino acids are depleted from NR conditioned stationary-phase media

**Figure 4:**
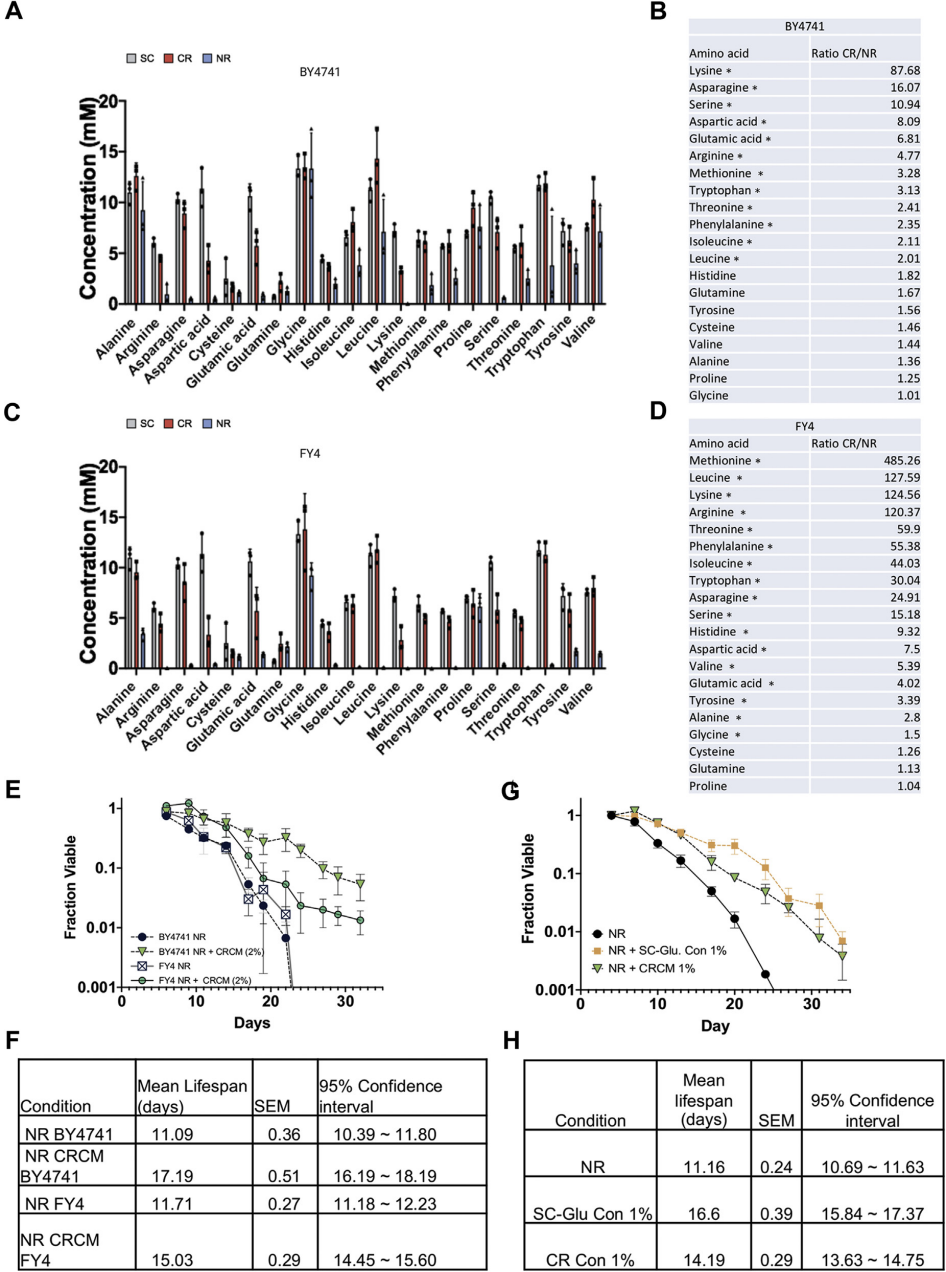
**CR conditioned media is enriched for multiple amino acids.***A*, quantification of amino acids in concentrated NRCM and CRCM from stationary-phase BY4741 cultures or the concentrated unconditioned SC media. Amino acids were separated with a ZipChip and quantified by mass spectrometry. Error bars indicate standard deviation (n = 3), and individual data points are displayed. *B*, amino acid abundance ratios between BY4741 CRCM and NRCM are sorted from highest to lowest. *C*, quantification of amino acids in concentrated NRCM and CRCM from stationary-phase FY4 cultures or unconditioned SC media. *D*, amino acid abundance ratios between FY4 CRCM and NRCM sorted from highest to lowest. Significant differences in panels B and D are indicated (∗*p* ≤ 0.05) using a Student *t*-test. *E*, quantitative CLS of BY4741 and FY4, each supplemented with CRCM isolated from BY4741 at 2% (vol/vol). *F*, mean CLS statistics from panel E calculated using OASIS 2. *G*, quantitative CLS of BY4741 supplemented with CRCM or unconditioned SC concentrate at 1% (vol/vol). *H*, Mean CLS statistics from panel G calculated using OASIS 2. CLS assays were run in biological triplicate.

We next profiled all 20 standard amino acids from BY4741 CRCM and NRCM concentrates, as well as unconditioned (fresh) SC media that was concentrated in the same manner (Fig. 4*A*). All but six amino acids (alanine, cysteine, glutamine, glycine, proline, and valine) were significantly depleted to varying degrees in NRCM concentrate, relative to unconditioned SC concentrate. CR strongly attenuated the depletion, indicating that amino acid levels were generally higher in CRCM than NRCM. L-serine is an excellent example of this relationship (Fig. 4*A*). The CR/NR abundance ratios for lysine, asparagine, and serine were each tenfold or higher in the CRCM (Fig. 4*B*), but still less than the level in unconditioned SC concentrate (Fig. 4*A*). Notably, the branched chain amino acids (BCAA) leucine and valine were significantly more abundant in CRCM concentrate than SC (Fig. 4, *A*–*B* and S3), with isoleucine trending in the same direction, suggesting that biosynthesis and release of these amino acids were induced by CR. This effect was lost in the prototrophic FY4 strain, suggesting the *leu2Δ* mutation in BY4741 could be a contributing factor. Otherwise, the pattern of CR rescuing amino acid depletion from the media was recapitulated with FY4, though stronger NR depletion rendered CR/NR ratios more extreme (Fig. 4, *C*–*D*). Accordingly, the CRCM concentrate isolated from prototrophic FY4 stationary-phase cultures was also effective at extending FY4 CLS (Fig. 4, *E*–*F*). Based on these results, we hypothesized that part of the CR longevity effect consisted of altering amino acid metabolism in such a way that prevented depletion from the media. Similarly, the short life span of NR cultures could be due to amino acid depletion. Higher amino acid levels in the CRCM concentrate could therefore explain why it was more effective than NRCM concentrate at extending CLS of NR cultures. Consistent with this interpretation, supplementing concentrated unconditioned SC media into NR cultures also extended CLS (Fig. 4, *G*–*H*).

### Supplementation of specific amino acids is sufficient to extend CLS

**Figure 5:**
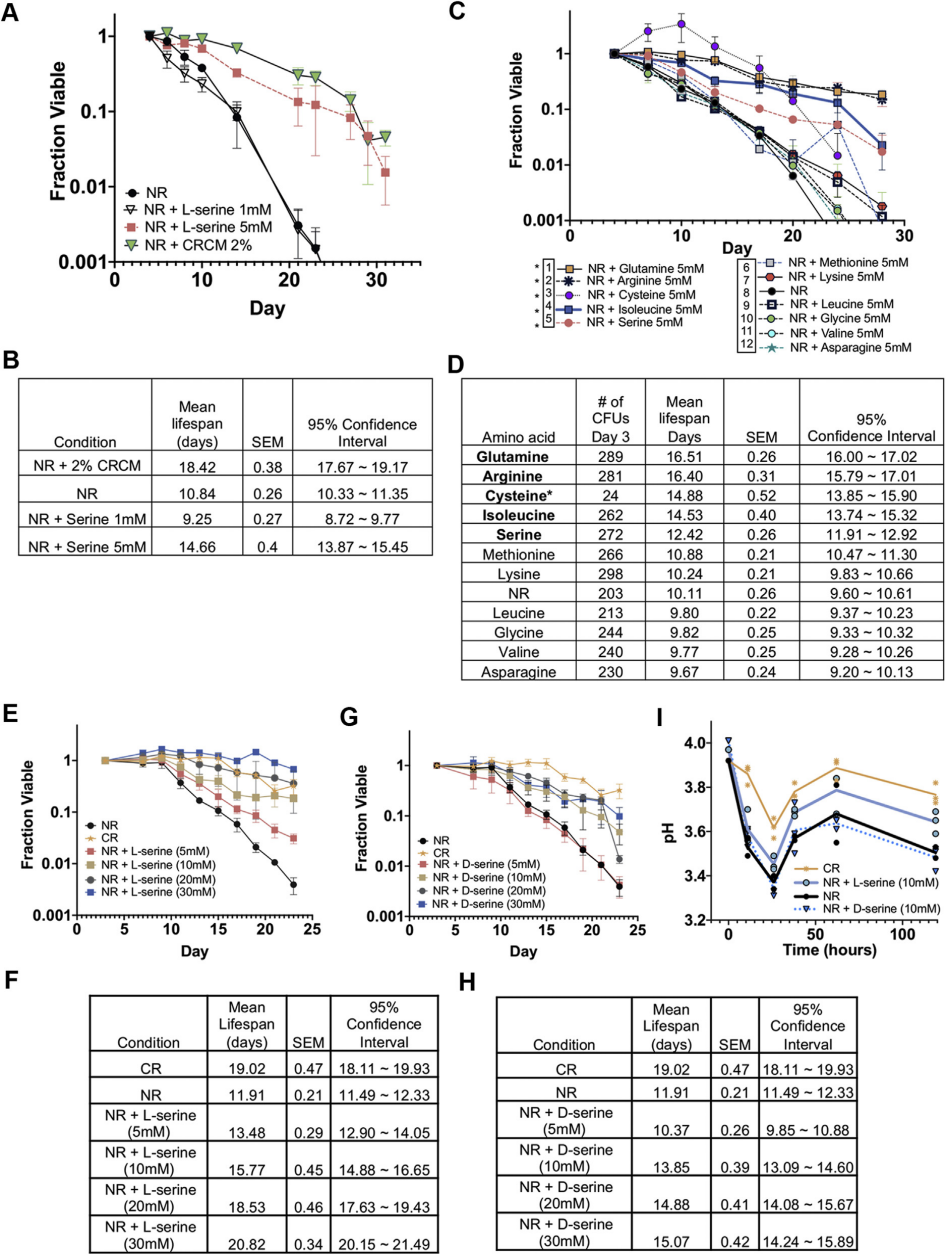
**L-Serine supplementation extends life span of NR cultures.***A*, CLS of nonrestricted BY4741 supplemented with 2% CRCM, 1 mM L-serine, or 5 mM L-serine. *B*, mean CLS statistics from panel A calculated using OASIS 2. *C*, CLS of nonrestricted BY4741 supplemented with 5 mM of each indicated amino acid. ∗Amino acids 1–5 significantly extended life span compared with the NR control (sample 8) (*D*) Mean CLS statistics from panel C calculated using OASIS 2. *E*, CLS of nonrestricted BY4741 supplemented with increasing concentrations of L-serine. CR indicates the glucose-restricted control samples. *F*, mean CLS statistics from panel E calculated using OASIS 2. *G*, CLS of BY4741 supplemented with increasing concentrations of D-serine. CR indicates the glucose-restricted control. *H*, mean CLS statistics from panel G calculated using OASIS 2. All CLS assays were run in triplicate. *I*, pH measurements of NR cultures supplemented with 10 mM L-serine or D-serine at the time of inoculation and then grown into stationary phase. CR is used as a positive control for suppressing hyperacidification. Individual data points are displayed for each time point.

Since most amino acids were depleted from stationary-phase NR cultures, we reasoned that one or more of them were critical for maintaining longevity. We initially focused on L-serine because the biosynthesis gene *SER1* was previously identified as a strong quantitative trait locus (QTL) for CLS in the BY4741 background (46), and *CHA1* expression was strongly induced by CRCM supplementation during the diauxic shift (Fig. 3*G*). The concentration of L-serine in our standard SC media is 1 mM (47, 48), so we tested the effect of supplementing an additional 1 mM or 5 mM L-serine into NR cultures at the time of inoculation (Fig. 5, *A*–*B*). In total, 5 mM L-serine significantly extended CLS, whereas 1 mM did not. To confirm that the L-serine effect was not specific to SC media, we also tested for CLS extension in a custom synthetic growth medium (HL) designed to support longevity that does not have ammonium sulfate as a nitrogen source (36, 49). BY4741 had significantly longer CLS in NR HL medium compared with SC medium, and 5 mM L-serine further extended it (Fig. S4, *A*–*B*). We next tested whether other amino acids could extend CLS at 5 mM (Fig. 5, *C*–*D*). Some amino acids did extend CLS, but not always as predicted based on abundance in the conditioned media. For example, L-asparagine had a similar depletion/enrichment profile as L-serine (Fig. 4*A*), but did not extend CLS when added back to NR cultures (Fig. 5, *C*–*D*). We also tested supplementation with 5 mM L-glycine, a component of one-carbon metabolism that can be derived from L-serine, but was not depleted from the NR media (Fig. 4*A*). L-glycine had no effect on CLS at this concentration (Fig. 5, *C*–*D*). L-cysteine supplementation at 5 mM dramatically slowed cell growth such that colony forming units (CFUs) were increasing until day 10, after which the decline in CLS was parallel to the NR control, suggesting a delay rather than a true extension of survival (Fig. 5, *C*–*D*). It is likely that each amino acid will have unique concentration dependence in regulating CLS.

### L-serine extends CLS through catabolic and noncatabolic pathways

An earlier study of cellular response to L-serine supplementation found that its uptake was linear with increasing extracellular concentrations up to at least 100 mM (45), suggesting to us that L-serine concentrations higher than 5 mM may induce stronger CLS extension. To test this idea, we supplemented NR cultures of BY4741 with 5, 10, 20, or 30 mM L-serine and observed progressively improved longevity with increasing concentration (Fig. 5, *E*–*F*). Survival with 30 mM L-serine was even slightly better than the CR control, showing minimal loss of viability during the experiment. A similar positive correlation between L-serine concentration and CLS was observed with FY4 (Fig. S4, *C–D*). To further examine whether improved CLS might result from catabolism of L-serine, we first supplemented NR cultures of BY4741 with the presumably nonutilized stereoisomer D-serine. We confirmed its inactivity by showing that 5 or 30 mM D-serine could not rescue the partial L-serine auxotrophic phenotype of a *ser2Δ* mutant in SC-serine media (Fig. S4, *E–H*). D-serine supplementation into BY4741 NR cultures had no effect on CLS at 5 mM (Fig. 5, *G*–*H*). It indistinguishably extended CLS at 10, 20, and 30 mM concentrations, but to a lesser extent than CR or the equivalent concentration of L-serine (Fig. 5, *G*–*H*). Based on these results, we hypothesized that L-serine catabolism is the primary mechanism of supporting CLS under NR conditions up to 5 or 10 mM, but additional noncatabolic mechanisms are involved at higher concentrations. An independent report concluded that exogenous amino acids, including L-serine, support CLS by preventing hyperacidification of the media (50). We therefore tested whether 10 mM L-serine and D-serine had the same pH buffering effect when supplemented into NR cultures. Compared with the CR positive control, 10 mM L-serine partially attenuated acidification as cultures transitioned to stationary phase (Fig. 5*I*), consistent with the earlier report (50). However, 10 mM D-serine supplementation had no effect on media pH across the same time course (Fig. 5*I*), despite its ability to extend CLS (Fig. 5*G*). We conclude that the pH buffering and CLS extension phenotypes caused by exogenous L-serine are most likely linked to its catabolism. D-serine, on the other hand, extends CLS through an unknown mechanism that could be shared by L-serine at higher concentrations.

### L-serine extends CLS through the one-carbon metabolism pathway

**Figure 6:**
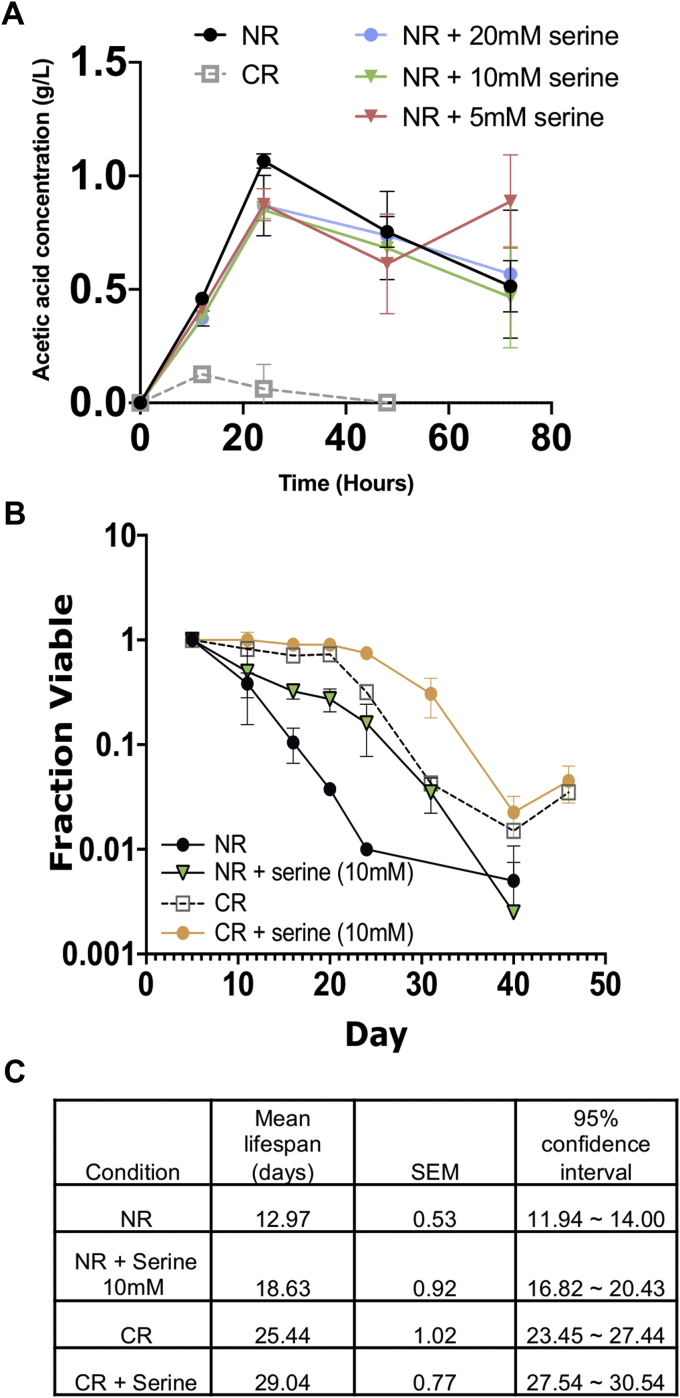
**CR and serine supplementation mediate longevity by independent mechanisms.***A,* Acetic acid content in the media of CR, NR, NR + L-serine (5, 10, 20 mM) cultures was measured over the first 72 h of the aging assays (mean ± SD, n = 3). *B,* CLS of BY4741 growing in the NR or CR media condition supplemented with 10 mM L-serine (n = 3). *C*, mean CLS statistics from panel B calculated using OASIS 2.

CR buffers the acidification of conditioned media by promoting consumption of acetate and acetic acid *via* Snf1/AMPK-dependent activation of gluconeogenesis and glyoxylate cycle gene transcription (16, 51). Since L-serine partially buffered against media acidification (Fig. 5*I*), we next tested whether supplementing L-serine into NR media also promoted acetic acid consumption. As shown in Fig. 6*A*, adding 5, 10, or 20 mM L-serine did not significantly reduce acetic acid levels in the NR media, indicating that pH buffering due to L-serine is unrelated to acetic acid accumulation or utilization. The result also implied that serine extends CLS through a mechanism different from CR. Indeed, L-serine further extended CLS when added to CR cultures (Fig. 6, *B*–*C*).

**Figure 7:**
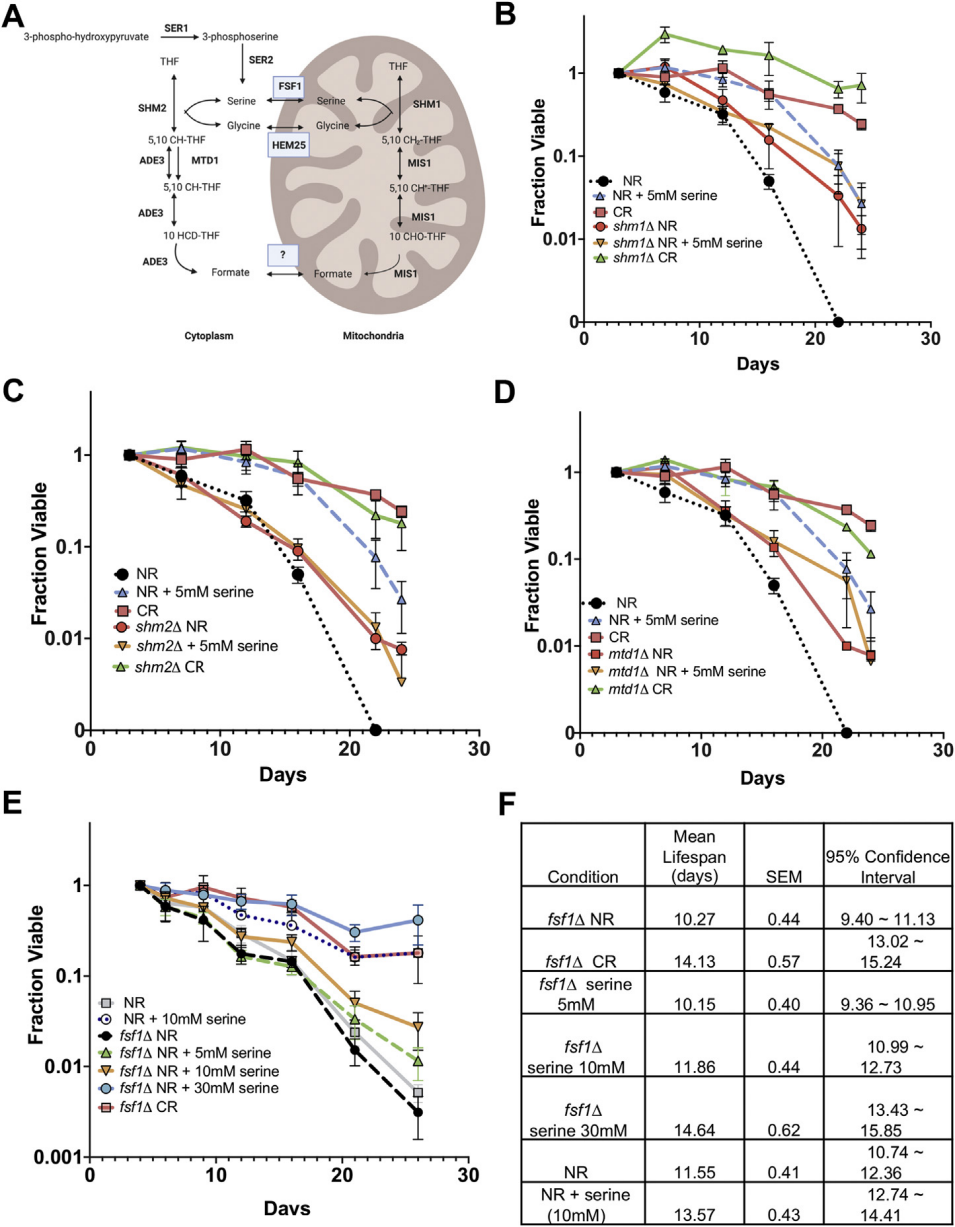
**L-serine extends CLS through the one-carbon metabolism pathway.***A*, schematic diagram of one-carbon metabolism is *Saccharomyces cerevisiae*. Enzymes that catalyze specific reactions are in bold. Transporters are boxed. *B*, CLS of BY4741 and *shm1Δ* mutant under NR and CR conditions or supplemented with 5 mM L-serine. *C*, CLS of BY4741 and *shm2Δ* mutant under NR and CR conditions or supplemented with 5 mM L-serine. *D*, CLS of BY4741 and *mtd1Δ* mutant under NR and CR conditions or supplemented with 5 mM L-serine. *E*, CLS of BY4741 and *fsf11Δ* mutant under NR and CR conditions or supplemented with 5 mM L-serine. *F*, mean CLS statistics from panel E calculated using OASIS 2. All CLS assays were run in triplicate.

Since L-serine is the predominant donor of carbon units to folate in one-carbon metabolism (52), we hypothesized its depletion would constrain this route of utilization in supporting CLS, which could explain extension by exogenous L-serine. If true, then mutations that impair one-carbon metabolism should attenuate the effect. The one-carbon metabolism pathway for *S. cerevisiae* is depicted in Fig. 7*A*, including serine hydroxymethyltransferases (SHMTs), Shm1 and Shm2, that interconvert L-serine and L-glycine in the mitochondria or cytoplasm, respectively. Shm2 is the major isozyme for converting L-serine to L-glycine and one-carbon units on tetrahydrofolate, whereas Shm1 is the predominant isozyme for the reverse reaction, though their relative activities are strongly influenced by nutrient availability and growth conditions (53). We therefore supplemented NR cultures of *shm1Δ* or *shm2Δ* mutants from the YKO collection with 5 mM L-serine to observe any effects on CLS. Without L-serine supplementation, the *shm1Δ* mutant showed moderate extension of mean CLS when compared with WT NR cultures (Fig. 7*B* and S5), while the *shm2Δ* mutant only showed modest improvements in survival at the later time points (Fig. 7*C* and S5). Importantly, both deletions prevented further CLS extension induced by 5 mM L-serine, but did not attenuate the strong positive life span effect of CR. A similar result was obtained for a strain lacking *MTD1* (Fig. 7*D* and S5), which encodes a cytoplasmic NAD^+^-dependent 5,10-methylenetetrahydrofolate dehydrogenase.

The mechanism of L-serine transport across the mitochondrial membrane for one-carbon metabolism was unknown until recently, when the Sideroflexin-1 protein SFXN1 was unexpectedly identified as the relevant transporter in human cells (54). Moreover, ectopic expression of the related yeast mitochondrial protein *Fsf1* rescued the L-serine transport and *de novo* purine synthesis defects of an SFXN1 mutant cell line, suggesting that Fsf1 has the same function in yeast cells. We therefore hypothesized that the CLS of *fsf1Δ* yeast cells would be unresponsiveness to L-serine supplementation, similar to the *shm1Δ*, *shm2Δ*, and *mtd1Δ* mutants. There was no indication of CLS extension for the *fsf1Δ* mutant under the NR condition (Fig. 7, *E*–*F*). However, the effects of 5 mM and 10 mM L-serine on CLS were blocked or attenuated, respectively (Fig. 7, *E*–*F*). CLS was still strongly extended by 30 mM L-serine, perhaps through alternative catabolic pathways such as deamination by Cha1, which is upregulated by high exogenous L-serine and the CRCM (Fig. 3*G*), or through the unknown D-serine-induced mechanism. We conclude that L-serine catabolism through the cytoplasmic and mitochondrial arms of the one-carbon metabolism pathway promotes chronological longevity/stationary-phase survival under NR conditions.

## Discussion

At the onset of this study, we hypothesized that CR may induce the production of one or more longevity factors, perhaps small molecules, peptides, or even proteins, that are released into the growth medium through either secretion from live cells or breakdown products from dying cells. Chromatography of CRCM and NRCM clearly indicated that the longevity factors were water-soluble small molecules, but we were surprised to find that the major differences between the two types of conditioned media were amino acids. Several unannotated compounds were more enriched in the CRCM, so at this time we cannot rule out the existence of other compounds with weaker effects on longevity.

### Amino acids as extracellular regulators of life span

CR in the context of this study consists of glucose restriction. However, dietary composition, not just overall caloric reduction, plays a critical role in modulating life span in multicellular organisms. In *Drosophila*, for example, lower dietary concentrations of yeast (amino acid source) or sugar generally improve life span, but moderate concentrations in combination are more optimal (55). Most cells in *Drosophila* or other multicellular organisms are not directly exposed to the environment, so they rely on specialized nutrient “sensing” cells that relay messages about nutrient availability, typically in the form of hormones (56). Unicellular organisms, on the other hand, must directly respond to nutrient fluctuations in the environment, making them dependent on both rapid detection and subsequent response to changes in nutrients. Yeast cells have multiple amino acid permeases that are under tight transcriptional and translational control, in order to properly regulate uptake (57). For example, when amino acids are scarce, translation of the *GCN4* mRNA is derepressed. Since Gcn4 is a transcriptional activator for these genes (58), this leads to transcriptional induction of most genes encoding amino acid biosynthetic enzymes, a regulatory process known as general amino acid control (GAAC) (59). The GAAC pathway also integrates with the TOR signaling pathway, which senses nitrogen availability (60), and links amino acid availability to life span regulation (22). Activation of GAAC generally reduces CLS, whereas suppression of GAAC extends CLS (61). This fits well with our finding that supplementing NR cultures with CRCM, which contains abundant amino acids, extends CLS, while NRCM that is amino acid depleted does not.

Common laboratory yeast strains such as W303, YPH499, and BY4741/BY4742 have several amino acid auxotrophies due to mutations in genes such as *HIS3*, *LYS2*, *LEU2*, *TRP1*, or *MET15*. Media containing standard concentrations of auxotrophy-complementing amino acids reduces the final biomass of cultures and shortens CLS, while excess amounts of these amino acids abrogates the aging phenotype (62). Amino acid uptake has also been genetically implicated in regulation of chronological aging. Chronological life span QTL analysis of outbred strains from a cross between S288C and a vineyard yeast strain revealed a polymorphism in the *BUL2* gene (63). *BUL2* encodes a subunit of an E3 ubiquitin ligase that controls trafficking of high-affinity amino acid permeases to the vacuole for degradation (64). Reduction in Bul2 function therefore stabilizes the permeases and increases intracellular amino acids, thus increasing TOR activity and shortening CLS. Most recently, availability of nonessential amino acids in the growth medium was shown to be important for chronological longevity (50). Specific amino acids were not critical, but rather the total amount of amino acids functioned to prevent hyperacidification of the growth medium (50). This scenario could also be at play with the numerous amino acids enriched in CRCM. In the case of L-serine, we found that it was indeed capable of buffering pH, but this effect was independent of accumulated acetic acid levels in the media.

Other specific amino acids have significant impact on life span as well. BCAA supplementation has been shown to extend CLS of *S. cerevisiae* (61) and *Caenorhabditis elegans* (65), consistent with the apparent biosynthesis of BCCA we observed under the CR condition (Fig. S3). However, BCAA restriction improves late-life health span and life span in *Drosophila* and mice (66, 67). Given such large differences in effects between species, this could reflect changes in amino acid balance, rather than direct effects due to BCAA levels (66, 67). Methionine restriction also extends life span in all model organisms tested thus far (68). It should be noted that BY4741 is auxotrophic for methionine due to the *met15Δ* mutation, indicating that this strain is already relatively long-lived compared with a strain that is *MET15*^+^ (23). Even with the *met15Δ* mutation, L-methionine or L-cysteine supplementation had little effect on CLS (Fig. 5, *C* and *D*). Furthermore, since CRCM and L-serine both extended CLS of FY4, the *met15Δ* mutation does not appear to be a major determinant for this cell extrinsic mechanism of life span regulation.

Less attention has been placed on L-serine within the aging research community. In addition to our work here and others showing that L-serine supplementation extends yeast CLS (50), L-serine was among the best amino acids at extending *C. elegans* life span when supplemented to the worms in a dose-dependent manner (69). L-serine supplementation to mice was also recently shown to reduce food intake, improve oxidative stress, and SIRT1 signaling in the hypothalamus of aging mice, though life span was not tested (70). Lastly, L-serine is also being studied as a possible neuroprotectant in the treatment of ALS and other neurodegenerative disorders (71, 72, 73). Despite these beneficial effects, supplementing with L-serine was reported to be proaging when the only other amino acids added to the media were those covering the auxotrophies (74). As with BCAA, these discrepancies could be due to the combination of auxotrophies and media content, which has been shown to be a major variable driving different CLS results from different labs (36, 39, 74).

### Why do amino acids accumulate in the conditioned media of CR cultures?

In the presence of sufficient glucose, *S. cerevisiae* cells actively suppress respiratory metabolism and biomass production through the tricarboxylic acid cycle (TCA) cycle, a phenomenon known as the Crabtree effect in yeast and the Warburg effect in cancer cells (75). When glucose becomes limiting, however, *S. cerevisiae* cells utilize oxidative metabolism over fermentative metabolism, resulting in elevated respiration and electron transport. Under such conditions, amino acids may be used to replenish TCA cycle intermediates through trans- and deamination reactions, a process called anaplerosis. Normally, in cells originally grown in 2% glucose (NR), glucose depletion triggers increased amino acid uptake that involves upregulation of permeases *via* TOR (76). Initial growth under CR (0.5% glucose) appears to generally reduce amino acid uptake as indicated by accumulation of amino acids we observe in the conditioned medium (Fig. 4, *A–D*) and instead prioritizes consumption of alternative carbon sources such as acetate (Fig. 6, *A*; (38, 48)), which yeast cells can convert into acetyl-CoA for TCA intermediate replenishment, or gluconeogenesis *via* the glyoxylate cycle (77). This likely better accommodates the increased storage of glycogen and trehalose induced by CR and associated with long-term cell survival in stationary phase (20). The higher cell densities (biomass) achieved by NR cultures instead place tremendous demand for synthesis of macromolecules associated with cell growth, such as nucleotides, lipids, and proteins, thus depleting amino acids from the media.

CR could also potentially make ammonium sulfate a preferred nitrogen source over the amino acids that are usually preferred under NR conditions. Ammonium sulfate has been shown to reduce CLS and is actually left out of the custom HL medium designed to optimize CLS (49, 78). Therefore, assimilation of the ammonium under CR could potentially extend CLS by reducing ammonium toxicity, similar to the CR-induced consumption of acetic acid (38, 48). Evidence for this mechanism comes from studies of amino acid preference during fermentation by wine yeasts (79). Of the 17 amino acids tracked, lysine was utilized the fastest, followed by a group of ten (Asp, Thr, Glu, Leu, His, Met, Ile, Ser, Gln, Phe) that were consumed quicker than ammonium sulfate and six (Val, Arg, Ala, Trp, Tyr, Gly) that were slower. Consistent with this hypothesis, lysine was the most depleted amino acid in BY4741 NR conditioned media and was partially rescued by CR (Fig. 4, *A* and *B*). Moreover, all of the fast-depleted amino acids in wine fermentation, except glutamine, were depleted under NR and rescued by CR (Fig. 4, *A* and *B*). Interestingly, extreme lysine harvesting from the media functions to channel NADPH normally used for lysine biosynthesis into glutathione production, providing an example of nutrient uptake triggering metabolism reconfiguration, not just to enabling cell growth (80).

### One-carbon metabolism in regulation of aging

Although multiple amino acids are more abundant in CRCM than NRCM, we focused on L-serine because the biosynthesis gene *SER1* is a QTL for CLS in the BY4741 background (46). L-serine is also a major entry point for the one-carbon metabolism pathway and a key component of the transsulfuration pathway (81), which has been implicated in longevity (82). The one-carbon metabolism pathway supports multiple cellular processes such as biosynthesis of purines, amino acid homeostasis (glycine, serine, and methionine), epigenetics through S-adenosylmethionine and chromatin methylation, and redox defense (83). However, few studies have directly linked one-carbon metabolism to the regulation of aging. In one aging-focused study, activation of naïve T cells from aged mice was attenuated because of a deficit in the induction of one-carbon metabolism enzymes (84).

In the current study, we identified a link between serine, one-carbon metabolism, and longevity. Specifically, we found that L-serine supplementation into NR cultures extended CLS in a manner dependent on the one-carbon metabolism pathway (Fig. 7), which we interpret as the one-carbon units donated from L-serine allowing cells to complete biosynthesis processes required to effectively enter quiescence. Of note, L-glycine, also a one-carbon donor, was not depleted from NR yeast cultures and had no effect on CLS when supplemented (Figs. 4*A* and 5*C*). In this sense, NR yeast cells may be similar to cancer cells that rely on exogenous serine, but not glycine, for proliferation (85). Overexpression of serine hydroxymethyltransferase SHMT2 is also associated with poor prognosis in cancer patients, while downregulating this enzyme suppresses tumorigenesis in human hepatocellular carcinoma (86).

Yeast cells lacking the mitochondrial serine hydroxymethyltransferase Shm1, or the NAD-dependent 5,10-methylenetetrahydrofolate dehydrogenase Mtd1, each displayed extended mean and maximum CLS. Cells lacking the cytoplasmic Shm2 enzyme also appeared to extend maximum CLS (Fig. 7). These results suggest that perturbing flux through the one-carbon metabolism pathway under NR conditions can influence long-term cell survival, perhaps by forcing metabolism toward gluconeogenesis and the glyoxylate cycle, thus mimicking CR. Interestingly, yeast replicative life span extension caused by deletion of the *RPL22A* gene was recently shown to correlate with reduced translation of one-carbon metabolism enzymes (87). Furthermore, deleting genes involved in one-carbon metabolism moderately extended replicative life span, similar to what we observed for CLS. Taken all together, we hypothesize that CR may also reduce flux through the one-carbon metabolism pathway, consistent with reduced serine consumption from the media and strong CR-induced CLS extension that occurred even in the *shm1Δ*, *shm2Δ*, or *mtd1Δ* mutants. The impact of these perturbations, which represent evolutionarily conserved and highly connected pathways, may depend on genetic and environmental context, and thus the yeast model is ideal for further systematic experimental characterization (88). Given the common effect of one-carbon metabolism on yeast RLS and CLS, and its strong conservation from yeast to mammals, future investigation of its roles in metazoan aging models is warranted.

## Experimental procedures

### Yeast strains and media

*S. cerevisiae* strains used in this study were BY4741 (*MAT***a** *his3Δ1 leu2Δ0 met15Δ0 ura3Δ0)*, FY4 (*MAT***a** prototrophic), and several deletion mutants from the Euroscarf yeast knockout (YKO) collection in the BY4741 background (89). Synthetic complete (SC) growth medium was used for all experiments except for the use of custom “human-like” HL media (36, 49). A recipe for SC media with individual amino acid concentrations is provided in Table S6 (47). Glucose was added to a final concentration of either 2.0% (non-restricted [NR]) or 0.5% (calorie restricted [CR]). To supplement amino acids, SC or HL medium was used containing 2% glucose (NR) and with amino acids added to a final concentration of 5, 10, 20, or 30 mM where indicated. Unless noted otherwise, all cultures (10 ml) were grown at 30 °C in 15 ml glass culture tubes with loose-fitting metal caps on a New Brunswick Scientific roller drum.

### Semiquantitative (spot) and quantitative assays for measuring CLS

To assess chronological life span, overnight 10 ml SC 2% glucose (NR) cultures were started from single colonies in triplicate. Next, 200 μl of the overnight cultures was used to inoculate fresh 10 ml cultures of the indicated SC medium conditions (NR, CR, or NR + amino acid). For each time point, 20 μl aliquots were then removed from cultures at the indicated times in stationary phase (starting at day 3) and serially diluted in tenfold increments with sterile water in 96-well plates. For semiquantitative spot assays, 2.5 μl of each dilution was spotted onto YPD 2% glucose agar plates as previously described (6). The plates were then digitally imaged on a gel documentation system after 2 days of colony growth, and the time points were compiled together to visualize the changes in viability over time. For the quantitative CLS assays, 2.5 μl of the 1:10, 1:100, and 1:1000 dilutions of each culture was spotted onto YPD plates that were then incubated at 30 °C for 18–24 h to allow for microcolony formation (16). We typically spot the three dilutions for 12 different cultures onto one YPD plate. Images of each dilution spot were captured on a Nikon Eclipse E400 tetrad dissection microscope at 30x magnification such that the entire spot fills the field of view. Microcolonies were then counted from the images either manually with a counting pen or automatically using OrganoSeg, a program originally developed for counting mammalian organoids in culture (90), which we have adapted for counting yeast colonies (Enriquez-Hesles *et al*., manuscript in preparation). At the end of each experiment, percent viability was calculated for each time point by normalizing to the first day of CFU measurements. Standard deviation error bars on the survival curve graphs were determined from three biological replicates. Statistical analysis was performed using the online program OASIS 2 (91), reporting mean life span (days), standard error of the mean (SEM), and 95th percentile confidence interval (95% CI) of the mean for each strain or condition. Q-HTCP analysis was performed as previously described (39).

### Chromatography

Fifteen BY4741 NR or CR cultures (10 ml SC each) were grown to saturation for 5 days at 30 °C in 18 mm glass test tubes with loose-fitting metal caps. Tubes were used instead of a single flask to maintain the same incubation conditions as our typical CLS assays. The glassware was acid washed with 0.1 N HCl before use. Cells were pelleted by centrifugation and the conditioned media pooled. The pooled media was then concentrated in a Büchi Rotavap from 150 ml down to approximately 2.5 ml (60-fold). The concentrates were centrifuged at 4000 rpm (2987 RCF) for 10 min in 15 ml conical tubes to remove any solid precipitates. Two milliliter of the clarified concentrate was loaded onto a 1 x 26 cm Sephadex G-10 column and fractionated with double-distilled water. Two milliliter fractions were collected by gravity flow in a Pharmacia fraction collector and filter sterilized through 0.22 μm syringe filters. The fractions were then added to new 5 ml CLS cultures at a 1:5 ratio (ml concentrate: ml culture) and semiquantitative CLS assays performed. NaCl (100 mM) was eluted through the column before and after the media concentrates to determine the gel size retention fractions as measured by electrical conductivity.

### Preparation of conditioned media concentrates for CLS and amino acid assays.

To collect and concentrate conditioned media for CLS assays and amino acid profiling, BY4741 or FY4 strains were grown in 150 ml SC cultures (with either 2% glucose or 0.5% glucose) at 30 °C for 5 days in a shaking water bath. The cultures were then centrifuged and the supernatants were condensed from 150 ml down to 15 ml (10-fold) using a Büchi Rotavapor-R apparatus, then filtered by passing through a 0.22 μm filter, and stored at –20 °C. For supplementation experiments, conditioned media concentrates (derived from NR or CR cultures) were added to 10 ml of SC-NR media to final concentrations of 1%, 2% (vol/vol), or higher where indicated.

### Metabolomics

BY4741 NR and CR cultures (10 ml SC each) were grown to stationary phase (day 5), then centrifuged in 15 ml disposable conical tubes (Falcon). The supernatant media was filter sterilized through 0.22 μm syringe filters and frozen at –80 °C. Untargeted metabolomics of conditioned media from 6 NR and 6 CR cultures was performed *via* gas chromatography/electron-ionization mass spectrometry (GC/ei-MS) in the Metabolomics Laboratory of the Duke Molecular Physiology Institute (DMPI), as described (92). Metabolites were extracted by the addition of methanol. Dried extracts were methoximated, trimethylsilylated, and run on a 7890B GC-5977B ei-MS (Agilent Corporation, Santa Clara, CA), with the MS set to scan broadly from *m/z* 50 to 600 during a GC heat ramp spanning 60–325 ºC. Deconvoluted spectra were annotated as metabolites using an orthogonal approach that incorporates both retention time (RT) from GC and the fragmentation pattern observed in MS. Peak annotation was based primarily on DMPI’s own RT-locked spectral library of metabolites, which is now one of the largest of its kind for GC/EI-MS. DMPI’s library is built upon the Fiehn GC/MS Metabolomics RTL Library (a gift from Agilent, their part number G1676–90000; (93)). Quantities from the mass spectrometry were normalized to OD_600_ of the cultures to account for cell density. In total, 160 metabolites were annotated based on matches with a spectral library. Another 115 metabolites were not matched in the library and remain unannotated (Table S1).

### Quantitative amino acid profiling

Conditioned NR and CR media from day 5 stationary-phase cultures was collected and concentrated with the Rotavap as described above. As a control, SC media without glucose was also concentrated and analyzed. Samples were submitted to the UVA Biomolecular Analysis Facility and then analyzed using a ZipChip system from 908 Devices that was interfaced with a Thermo Orbitrap QE HF-X Mass Spectrometer. Samples were prepared by diluting 10 μl with 490 μl of LC-MS grade water, which was then further diluted 1:10 with 90 μl of the ZipChip diluent (908 Devices Inc, P/N 810-00168). The samples were loaded onto ZipChip HR Chip (908 Devices Inc, P/N 810-00194) for analysis. The following ZipChip analysis settings were utilized: Field strength: 500 V/cm, Injection volume: 7 nl, Chip Type: HR, BGE: Metabolite, Pressure assist: Enable at 7 min, Run time: 10 min, MS setting (Thermo Orbitrap QE HF-X), m/z range: 70–500, Resolution: 15,000, 1 microscan, AGC target: 3E6, Max ion injection time: 20 ms, Inlet capillary temperature: 200 °C, S Lens RF: 50.

### RNA analysis

Cells from NR overnight cultures were inoculated into 75 ml of fresh NR SC medium that was supplemented with 1.5 ml of concentrated conditioned media (CRCM or NRCM) or 1.5 ml of sterile water as a control. The starting OD_600_ was 0.05 in 250 ml Erlenmeyer flasks. Cultures were grown at 30 °C in a New Brunswick water bath shaker. For the log-phase condition, 50 ml of the samples was collected at OD_600_ of 0.2. Equivalent numbers of cells were collected from smaller aliquots harvested at 24 h and 96 h. Total RNA from three biological replicates was isolated using the hot acid phenol method and then processed into Illumina DNA sequencing libraries as previously described (51), with slight modifications. Briefly, total RNA was treated with DNase I for 10 min at 37 °C and then measured for concentration and quality with an Agilent Bioanalyzer. PolyA mRNA selection was performed on 5 μg of the DNase-treated total RNA with the NEBNext Poly(A) mRNA magnetic isolation module (E7490). DNA sequencing libraries were then generated with the NEBNext Ultra Directional RNA library Prep kit for Illumina (E7420). Libraries were sequenced on an Illumina NextSeq 500 by the UVA Genome Analysis and Technology Core (GATC). Sequencing files are available at GEO (accession number GSE151185). Sequencing reads were mapped to the sacCer3 genome using bowtie2 with default settings (94). We preprocessed sequencing data from the UVA GATC and analyzed differential gene expression in R using DESeq2 (95).

### Acetic acid measurements

In total, 100 μl aliquots were taken at designated time points from standard 10 ml CLS cultures. Cells were pelleted by centrifugation at 2500 rpm at 4 °C, and 50 μl of supernatant was removed and stored at −80 °C, until further analysis. Acetic acid concentration for each sample was then later determined using an Acetic Acid Kit (Biopharm AG) per manufacturer's instructions. The acetic acid concentrations and standard deviations provided are an average of three biological replicates for each condition and reported as g/L.

## Data availability

All data from this study except raw Illumina RNA-seq data sets are contained within the article, including Supplemental Information. Sequencing files are deposited in the NCBI Gene Expression Omnibus (GEO) database at accession number GSE151185.

## Conflict of interest

The authors declare that they have no conflicts of interest with the contents of this article.
