# Supplementary material for: A cell-nonautonomous mechanism of yeast chronological aging regulated by caloric restriction and one-carbon metabolism
Source: J Biol Chem. 2020 Dec 2;296:100125. doi: 10.1074/jbc.RA120.015402 (PMC7949035; doi:10.1074/jbc.RA120.015402)
Supplement: Figures S1–S5 [file mmc1.pdf]

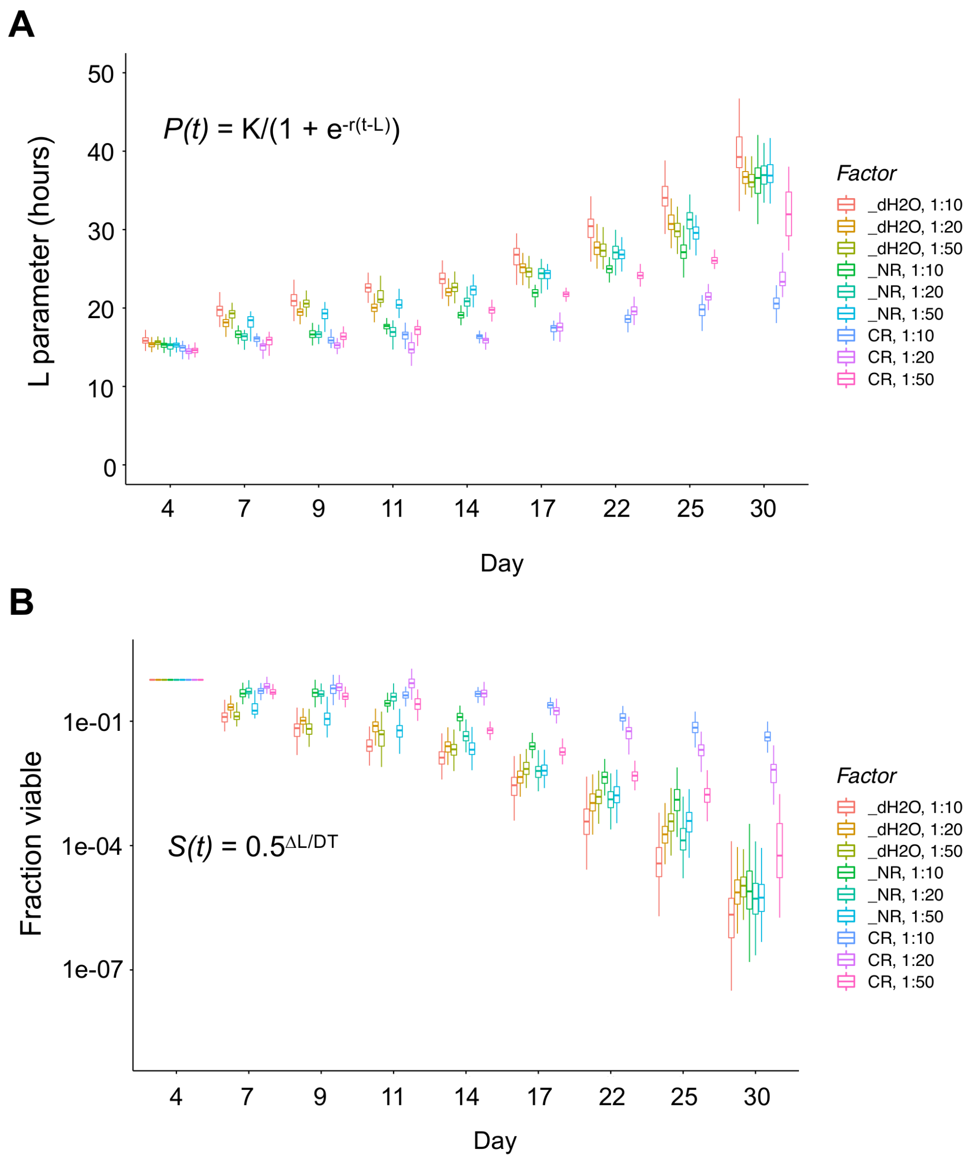

Figure S1. Longevity factors are enriched in conditioned media from glucose-restricted yeast cultures. (A) Quantitative high throughput cell array phenotyping (Q-HTCP) assay for detecting longevity factor activity. BY4741 was inoculated into SC NR (2% glucose) media, in 50 µl volumes in 384-well 'Aging Arrays'. Cultures were supplemented with various dilutions of 10X-concentrated conditioned media from 5-day old CR (0.5% glucose) or NR (2% glucose) cultures or treated with an equal volume of water, as indicated in the legend. At the indicated days, cells from the aging arrays were printed onto YPD Growth Array plates, and L values in hours were obtained. A strong dose response to longevity factors in CR conditioned media was detected, as indicated by lower L values. Each box in the plot represents the distribution of 96 replicate cultures. The equation used calculate L is provided. **(B)** The L value was also converted to fraction viable at each time point using the provided equation and presented as a more traditional survival curve.

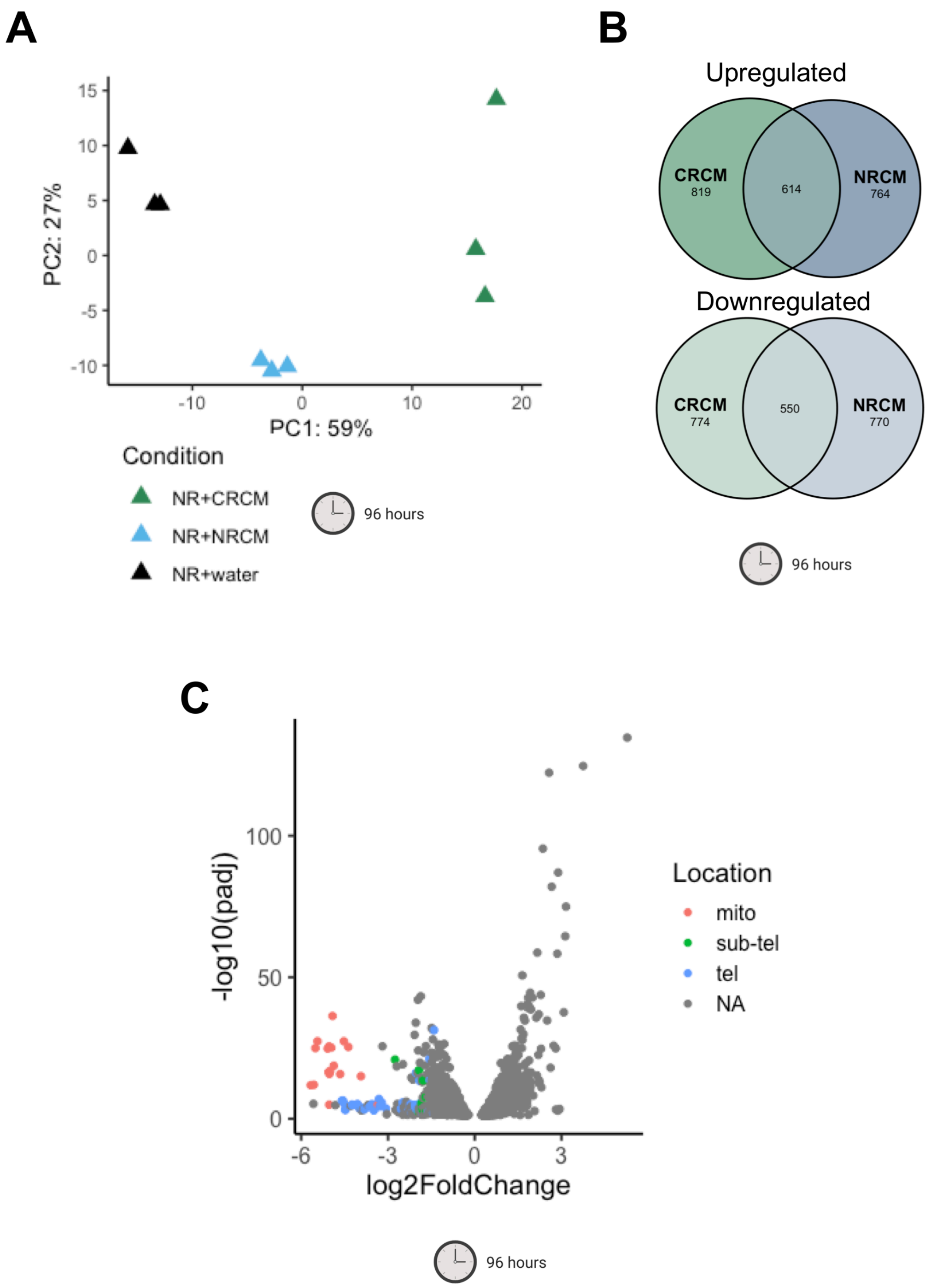

Figure S2. RNA-seq analysis of gene expression changes induced by CRCM and NRCM. (A) Principal component analysis of RNA-seq samples collected at 96 hours. **(B)** Venn diagram of differentially expressed genes (up or down; FDR<0.05) for NRCM or and CRCM compared to the NR control at 96 hrs. **(C)** Volcano plot displaying differential expressed genes compared between NR + CRCM and NR control samples at 96 hrs. The vertical axis (y-axis) corresponds to the p-adjusted value and the horizontal axis (x-axis) displays the log2-fold change value. Red, green, and blue denotes genes located in the mitochondrial genome, sub-telomeric, or telomeric regions, respectively.

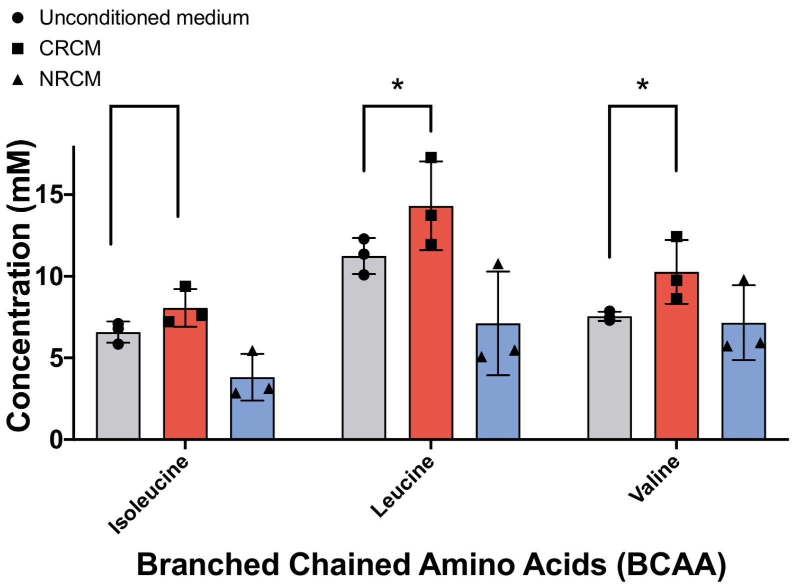

Figure S3. Branched chain amino acid levels in concentrated CRCM or NRCM. L-isoleucine, L-leucine, and L-valine levels were measured in concentrates of CRCM, NRCM, or unconditioned SC media. Error bars indicate standard deviation from three biological replicates. Significant increases in CRCM over the starting SC media are indicated by an asterisk ($p<0.05$, student's t-test).

A C

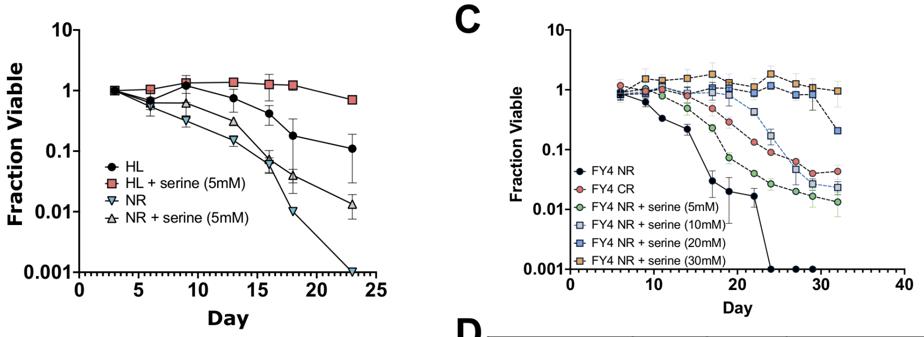

B

Condition	Mean Lifespan (days)	SEM	95% Confidence Interval
HL	13	0.40	12.44 ~ 14.01
HL + serine 5mM	21.38	0.32	20.75 ~ 22.00
NR	8.88	0.25	8.38 ~ 9.38
NR + serine 5mM	11.49	0.33	10.84 ~ 12.14

D

Condition	Mean Lifespan (days)	SEM	95% Confidence Interval
CR FY4	16.94	0.49	15.99 ~ 17.89
NR FY4	16.88	0.41	16.07 ~ 17.68
NR + serine FY4	12.2	0.32	11.57 ~ 12.82
NR + serine 10mM FY4	19.43	0.4	18.65 ~ 20.21
NR + serine 20mM FY4	26.48	0.49	25.52 ~ 27.44
NR + serine 30mM FY4	27.25	0.41	26.45 ~ 28.04

E F G H

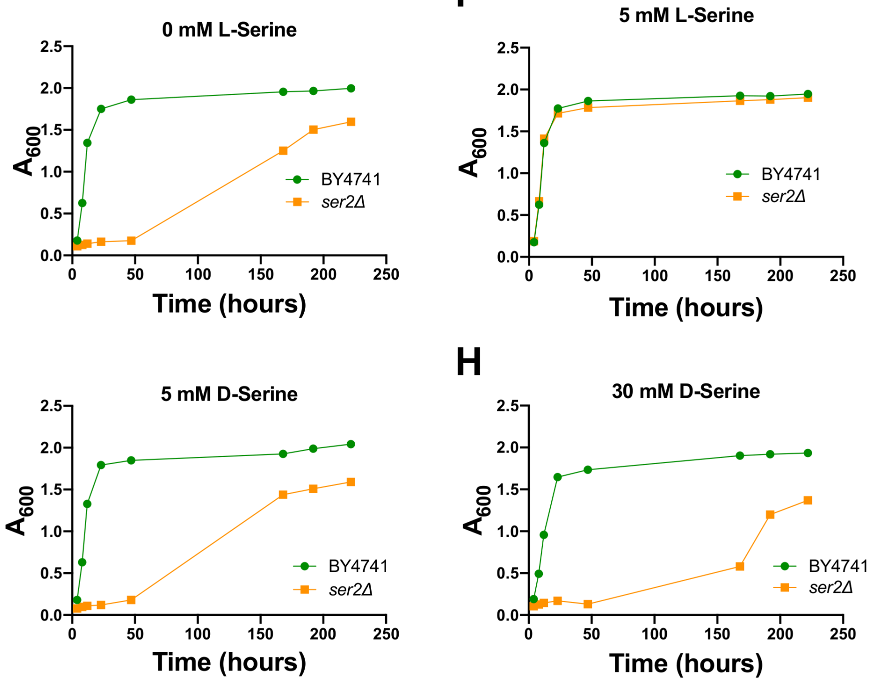

Figure S4. Analysis of L-serine and D-serine effects on CLS and cell growth. (A) CLS of BY4741 cells grown in SC (NR) media or custom Human-Like (HL) medium supplemented with 5 mM L-serine. **(B)** Mean CLS statistics from panel A, calculated using OASIS 2. **(C)** CLS of prototrophic strain FY4 in SC (NR) media supplemented with L-serine at the indicated concentrations. FY4 was also grown in SC (CR) media as a control. **(D)** Mean CLS statistics from panel C, calculated using OASIS 2. **(E-H)** Growth curves of BY4741 and *ser2*Δ mutant in SC-serine media supplemented with the indicated concentrations of L-serine or D-serine (mean, n=2).

Condition	Mean Lifespan (days)	SEM	95% Confidence Interval
NR	11.24	0.53	10.20 ~ 12.27
CR	17.14	0.51	16.15 ~ 18.14
NR + 5mM serine	17.71	0.56	16.61 ~ 18.81
*shm1*Δ NR	14.93	0.34	14.26 ~ 15.60
*shm1*Δ CR	16.72	0.44	15.85 ~ 17.58
*shm1*Δ NR + 5mM serine	13.08	0.58	11.94 ~ 14.22
*shm2*Δ NR	11.26	0.55	10.18 ~ 12.34
*shm2*Δ CR	18.64	0.59	17.50 ~ 19.79
*shm2*Δ NR + 5mM serine	10.69	0.45	9.82 ~ 11.57
*mtd1*Δ NR	13.43	0.40	12.65 ~ 14.21
*mtd1*Δ CR	17.05	0.52	16.04 ~ 18.07
*mtd1*Δ NR+ 5mM serine	13.92	0.42	13.09 ~ 14.75

Figure S5. Statistical analysis of CLS assays for one-carbon metabolism deletion mutants. Statistics for CLS assays presented in Figure 7, panels B, C, and D. The mean CLS, SEMs, and 95% confidence intervals were calculated using OASIS 2.

.
